# Health Insurance and Trauma Outcomes: The Impact of Coverage on Survival and Recovery in Craniomaxillofacial Injuries

**DOI:** 10.7759/cureus.105543

**Published:** 2026-03-20

**Authors:** Daniela Bresciani, Gabriela Pomales Díaz, Natalia Yordan Fernandez, Deyna Morales Carrasquillo, Cristhian G Negron Rodriguez, Jayson Cotto, Odaly S Balasquide, Guillermo S López, Angel J Carrero Agron, Juan C Berríos Cordero, Luis Barreto Hollady, Alejandra Acevedo Muñiz, Corally López Vega, Raúl Y Ramos-Sánchez, Angel Rivera Barrios

**Affiliations:** 1 Department of General Surgery, NewYork-Presbyterian Queens, New York, USA; 2 Department of General Surgery, Baylor University Medical Center, Dallas, USA; 3 Department of General Surgery, University of Puerto Rico, Medical Sciences Campus, San Juan, PRI; 4 Department of General Surgery, Universidad Central del Caribe, Bayamón, PRI; 5 Department of Otolaryngology - Head and Neck Surgery, University of Puerto Rico, Medical Sciences Campus, San Juan, PRI; 6 Department of Plastic Surgery, University of Puerto Rico, Medical Sciences Campus, San Juan, PRI

**Keywords:** craniomaxillofacial trauma, health disparities, health insurance, puerto rico, trauma outcomes

## Abstract

Introduction

Health disparities, particularly those related to insurance status, significantly impact trauma outcomes. Craniomaxillofacial (CMF) trauma patients without insurance often experience worse outcomes due to limited access to care and delayed treatment. This study examines the effects of insurance coverage on outcomes such as length of stay (LOS), survival, and surgical site infections (SSIs) among CMF trauma patients in Puerto Rico.

Methods

An institutional review board-approved retrospective analysis was conducted on 1,165 CMF trauma patients treated at the Puerto Rico Medical Center’s Department of Trauma from January 2018 to October 2022. Patient demographics, trauma type, insurance coverage, and outcomes were abstracted. Patients were categorized by insurance status: government-only, private-only, government + private, and uninsured. Statistical analyses, including ANOVA, chi-square tests, and t-tests, were performed to assess the relationships between insurance status and LOS, survival, operative status, and SSI.

Results

Most patients (n = 849, 72.9%) had government insurance only, 60 (5.2%) had government + private, 141 (12.1%) had private only, and 99 (8.5%) were uninsured. Motor vehicle accidents were the leading cause of injury (n = 575, 49.3%). Uninsured patients had shorter LOS (mean 12.43 days) compared to those with government (20.30 days) or private insurance (16.88 days; p = 0.02). There were 84 (9.9%) deaths in the government-only insurance group, 21 (14.9%) in the private-only insurance group, 10 (16.7%) in the government + private insurance group, and 20 (20.2%) in the uninsured group (p = 0.02). While uninsured patients were less likely to undergo surgery, there were no statistically significant differences in operative rates (p = 0.355) or SSI among insurance groups (p = 0.847).

Conclusions

This study highlights potential disparities in outcomes for CMF trauma patients in Puerto Rico based on insurance status. Government-insured patients had longer LOS but lower mortality, while uninsured patients experienced shorter stays and higher mortality, suggesting potential undertreatment. Expanding insurance coverage and improving follow-up care for uninsured populations could help address these disparities. Further research is needed to identify systemic factors, identify reasons for lack of insurance, and inform equitable trauma care policies.

## Introduction

Health disparities significantly influence patient outcomes, particularly in emergencies like craniomaxillofacial (CMF) trauma [1,2]. Insurance status is a critical determinant of care quality and survival, with uninsured patients consistently experiencing poorer outcomes than their insured counterparts [3]. Limited access to primary care among uninsured individuals often leads to undiagnosed comorbidities and delayed treatment, exacerbating their vulnerability [1]. For example, Chun et al. [4] found that uninsured patients have the highest trauma mortality rates, followed by those with Medicaid, Medicare, and private insurance. These disparities reflect poorer initial health status [5,6] and reduced chances of survival and health-related quality of life, regardless of age or injury severity [5]. Uninsured patients also undergo fewer diagnostic imaging studies, potentially due to financial limitations, which may delay diagnosis and treatment and heighten the risk of complications [7]. Additionally, shorter hospital stays among uninsured patients highlight decreased resource utilization, often hindering recovery and positive outcomes [8].

According to the 2020 Census, Puerto Rico’s health insurance coverage profile differs significantly from that of the US overall. In Puerto Rico, 43.9% of residents were insured through Medicaid, compared to 18.9% in the US; 14.3% were covered by Medicare, versus 18.9% nationally; 6.21% were uninsured, compared to 8% in the US; and only 34.6% had private insurance, in contrast to 65.4% across the US [9]. These figures highlight the distinct distribution of health insurance coverage among Puerto Rico residents. While public programs such as Medicare, Medicaid (known locally as Vital), and the Automobile Accident Compensation Administration (ACAA) provide critical services, a substantial portion of the population remains uninsured, pointing to ongoing systemic barriers to healthcare access. By 2022, 13.9% of Puerto Ricans were enrolled in Medicare, and approximately 1.5 million relied on Medicaid for coverage [10]. ACAA, a government-run program that covers medical expenses related to motor vehicle accidents (MVAs), further illustrates the unique landscape of insurance options within Puerto Rico’s healthcare system [11].

Although these insurance programs play a vital role in mitigating disparities, prior research has demonstrated that coverage gaps significantly impact trauma outcomes. However, there is limited data on how insurance status specifically affects CMF trauma outcomes in Puerto Rico, a resource-constrained setting with unique healthcare challenges. This study aims to address this gap by comparing (1) length of stay (LOS); (2) survival; and (3) operative rates and surgical site infections (SSIs) across insurance groups of CMF trauma patients.

## Materials and methods

This study was approved by the University of Puerto Rico, Medical Sciences Campus Human Research Subjects Protection Office Institutional Review Board (approval 2303084772R001). This was a retrospective analysis of patients who were treated for CMF traumas at the Department of Trauma of Administración de Servicios Médicos de Puerto Rico (ASEM) at the Medical Center of Puerto Rico from January 2018 to October 2022. Patients were identified using the Institutional Trauma Registry database. Eligible cases were identified using the ICD-10 diagnostic codes corresponding to CMF fractures recorded in the registry (Appendix A). All patients diagnosed with CMF trauma during this time period were included. CMF fractures were classified based on CT imaging findings documented in radiology reports and medical records. Data were abstracted from the trauma registry and electronic medical records by the study investigators and included patient demographics (age and gender), mechanism of injury, type of CMF fracture, insurance coverage, and clinical outcomes. Outcomes of interest included survival, hospital LOS, operative management status, and SSI. All extracted data were anonymized prior to analysis to ensure patient confidentiality.

Patients were categorized according to health insurance coverage: government insurance only, private insurance only, government + private insurance, and uninsured. Government insurance encompassed Medicare, Vital, and ACAA plans. Survival status was classified as alive at discharge or deceased during hospital admission. LOS was defined as the number of days from admission until discharge. Operative status refers to whether the patient received surgery for the treatment of the CMF trauma, and SSI was determined by intrahospital development of infection at the site of a surgically treated CMF trauma.

ANOVA was employed to compare differences in means of continuous variables between insurance groups, while the relationship between binary categorical variables and quantitative variables was analyzed with a t-test. A chi-square test of independence was used to determine statistical significance among different categorical variables. Statistical significance was determined at a p-value of ≤ 0.05.

## Results

Patient characteristics

**Figure 1:**
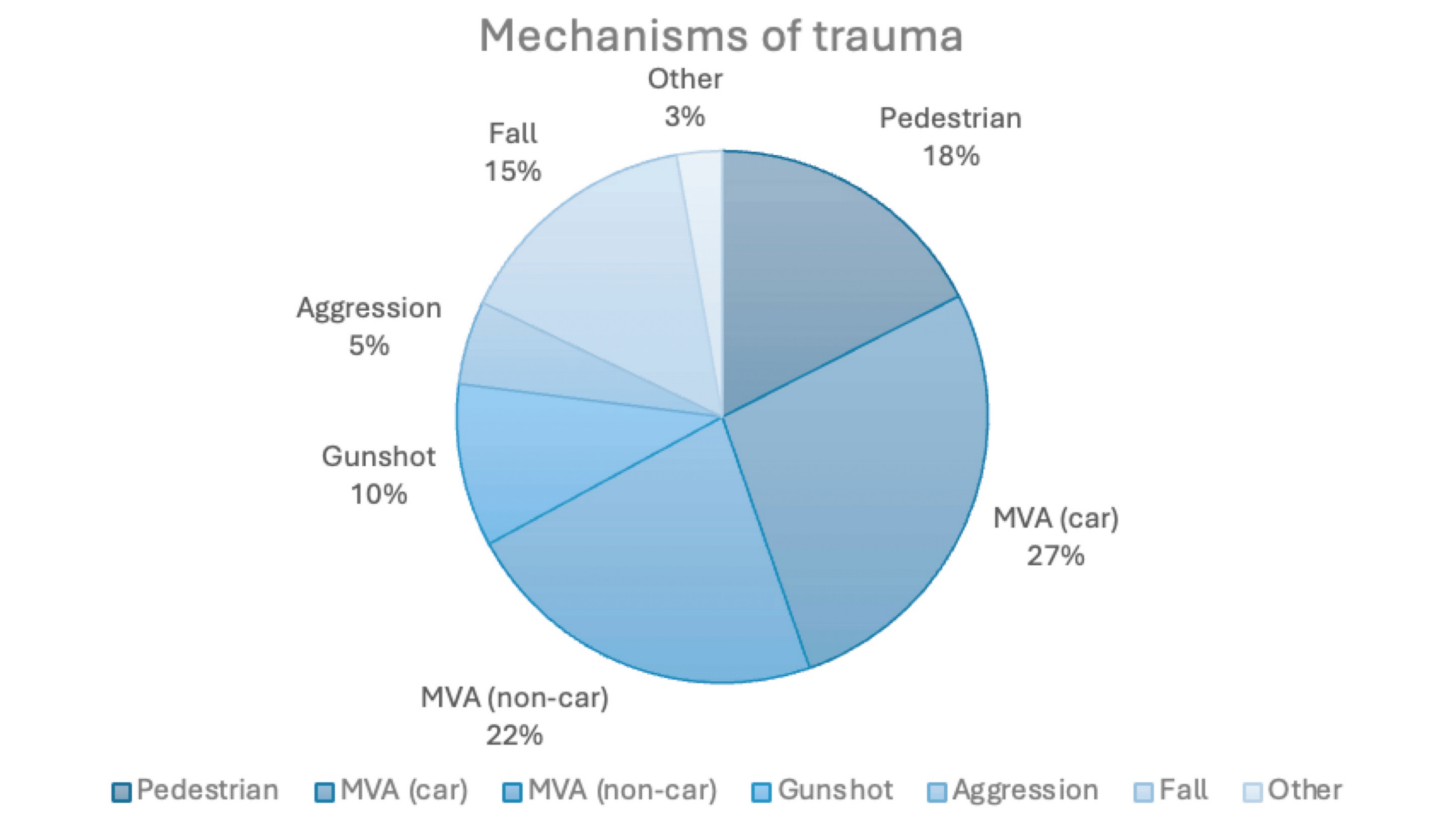
Mechanisms of trauma among patients MVA, motor vehicle accident

A total of 1,165 patients were treated for CMF traumas at the ASEM at the Medical Center of Puerto Rico from January 2018 to October 2022. Patient characteristics are detailed in Table 1. The mean age of the study population was 40.5 years (range: 0-100 years). Males predominated (n = 965, 82.8%), while there were 200 (17.2%) females. In terms of CMF trauma types, 494 (42.4%) patients sustained facial fractures, 64 (5.5%) had cranial fractures, 297 (25.5%) presented with both facial and cranial fractures, and 310 (26.6%) experienced non-fracture CMF trauma, such as soft tissue injuries requiring treatment. Patients’ mechanism of trauma is summarized in Figure 1. MVAs were the most common mechanism of trauma, with 27% of injuries resulting from car-related MVAs and 22% from non-car MVAs.

**Table 1:** Patient characteristics

Patient characteristics	N	%
Age (years)		
Mean	40.5	-
Range	0-100	-
Gender		
Female	200	17.2%
Male	965	82.8%
Trauma type		
Cranial fracture	64	5.5%
Facial fracture	494	42.4%
Cranial and facial fractures	297	25.5%
Non-fracture injury	310	26.6%

Table 2 presents the distribution of health insurance coverage among the patients. The majority, 849 (72.9%), were covered only by government insurance programs, with most patients being insured through the Vital program (n = 523, 61.6%), with or without additional government insurance. A further 60 (5.2%) of all patients had a combination of government + private insurance, most commonly private + ACAA (n = 31, 51.7%), with or without additional government insurance. Private insurance alone covered 141 (12.1%) patients, while 99 (8.5%) were uninsured. Insurance status was not recorded for 16 (1.4%) patients.

**Table 2:** Health insurance distribution ACAA, Automobile Accident Compensation Administration

Health insurance	N	%
Government	849	72.9%
Vital	394	46.4%
Medicare	24	2.8%
ACAA	296	34.9%
Vital + Medicare	12	1.4%
Vital + ACAA	114	13.4%
Medicare + ACAA	6	0.7%
Vital + Medicare + ACAA	3	0.4%
Government + private	60	5.2%
Private + Vital	13	21.7%
Private + Medicare	6	10.0%
Private + ACAA	31	51.7%
Private + Vital + Medicare	2	3.3%
Private + Vital + ACAA	4	6.7%
Private + Medicare + ACAA	2	3.3%
Private + Vital + Medicare + ACAA	2	3.3%
Private only	141	12.1%
Uninsured	99	8.5%
Not recorded	16	1.4%

Operative status and SSI

Among all patients, 213 (18.3%) underwent a surgical intervention for their CMF trauma. Specifically, 176 (20.7%) of those with government insurance underwent surgery, compared to 20 (14.2%) of those with private insurance, 11 (18.3%) of those with private and government insurance, and 17 (17.2%) of those uninsured (p = 0.355).

Among those who underwent surgery, 14 (8.4%) of those with government insurance, one (5.3%) with private insurance, one (9.1%) with private and government insurance, and two (12.5%) uninsured patients developed an SSI. However, these differences did not reach statistical significance (p = 0.847).

Survival

**Figure 2:**
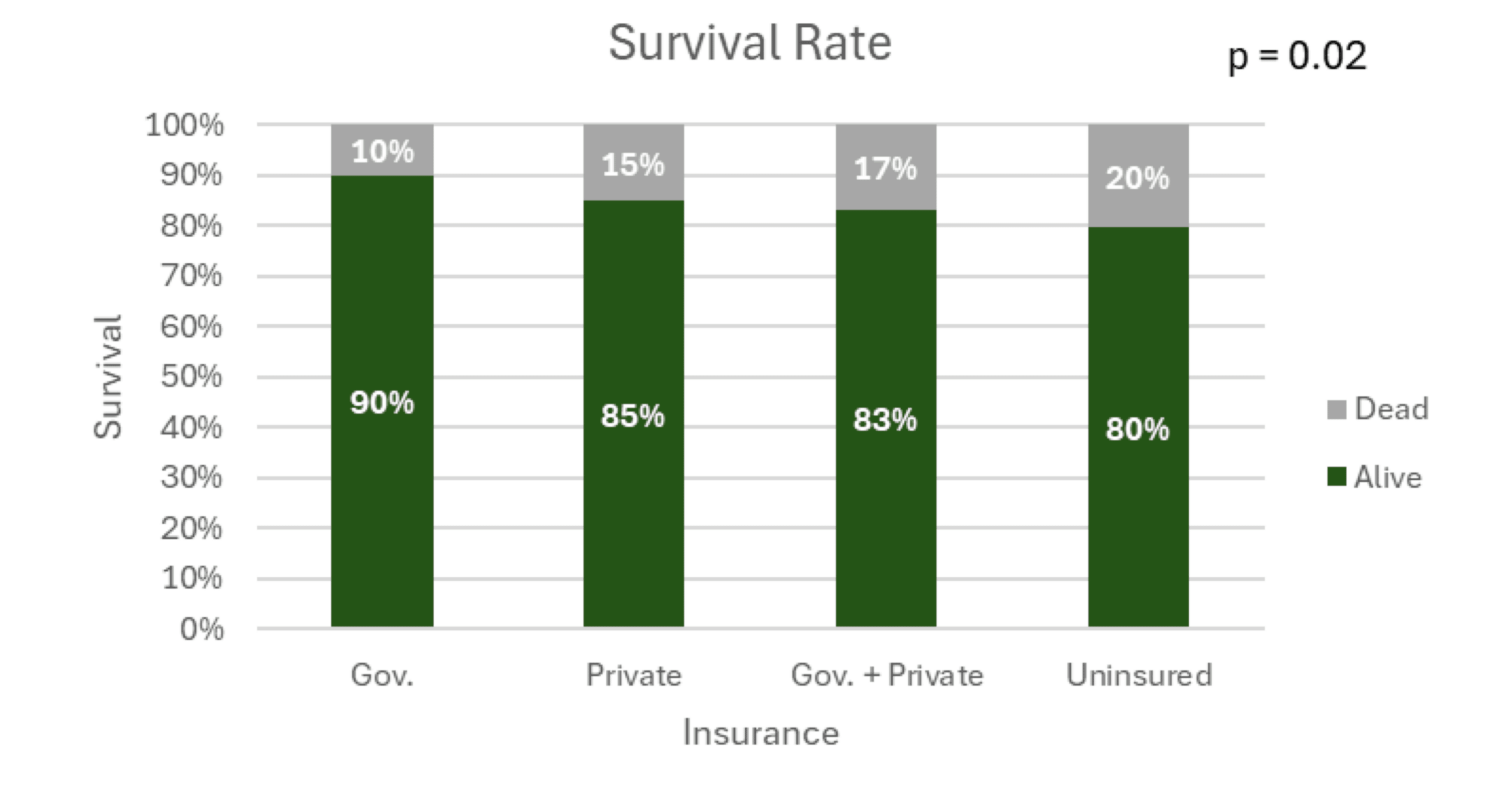
Survival rates by type of insurance Differences were statistically significant. Gov., government

Overall, 1,028 (88.2%) patients survived, and 137 (11.8%) died during their admission. Survival and mortality rates are depicted in Figure 2. There were 84 (9.9%) deaths in the government-only insurance group, 21 (14.9%) in the private-only insurance group, 10 (16.7%) in the government + private insurance group, and 20 (20.2%) in the uninsured group. The chi-square test showed a statistically significant association between health insurance status and patient survival (p = 0.02).

LOS

The overall mean LOS for all patients was 19.3 days (range: 0-232 days), while for survivors, the mean LOS increased to 20.5 days (range: 2-198 days). Nonetheless, t-test analysis indicated no significant difference in LOS between survivors (mean = 19.33 days, SD = 22.74) and non-survivors (mean = 20.28 days, SD = 35.67; p = 0.76). Similarly, no significant difference was found between operative and nonoperative groups in terms of LOS (operative: mean = 21.57 days, SD = 21.13; nonoperative: mean = 19.21 days, SD = 25.86; p = 0.21).

**Figure 3:**
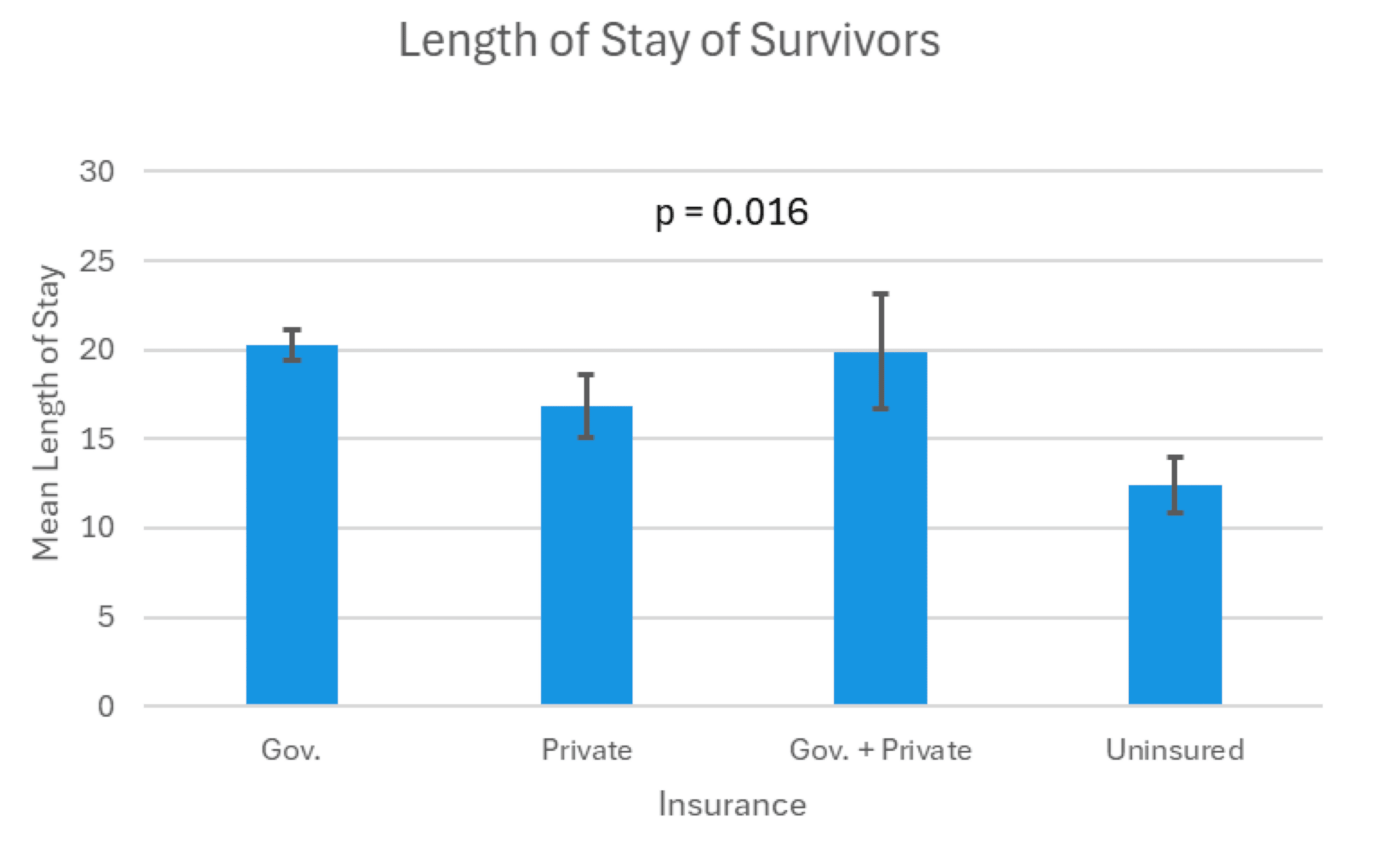
Mean LOS by type of insurance Error bars represent standard error. Differences were statistically significant. Gov., government; LOS, length of stay

Among survivors, patients with government-only insurance had the longest mean LOS at 20.30 days (SD = 23.578), followed by those with both government + private insurance at 19.9 days (SD = 22.78). Patients with private insurance had a mean LOS of 16.88 days (SD = 19.25), while uninsured patients had the shortest LOS, averaging 12.43 days (SD = 14.13). ANOVA results revealed a statistically significant difference in LOS across the health insurance groups (p = 0.02) (Figure 3).

## Discussion

Our study highlights significant disparities in outcomes for CMF trauma patients based on insurance status, with notable implications for Puerto Rico’s healthcare system. Consistent with prior trauma research, young, male patients comprised the majority of CMF trauma cases, reflecting the well-documented higher incidence of trauma among this group [12,13].

A substantial proportion of patients (78.1%) relied on government insurance, underscoring its critical role in Puerto Rico, where nearly half the population depends on public healthcare programs like Medicaid (Vital) due to economic constraints [14]. Compared to the US mainland, where public health coverage encompasses approximately 36.1% of the population [15], this reliance reveals systemic barriers to private insurance access on the island. These barriers likely contribute to the trauma-related healthcare disparities observed in Puerto Rico [16]. Additionally, these facts raise questions about the actual status of multiple insurance use (i.e., both governmental and private).

Our analysis of LOS for survivors showed significant differences across insurance groups. Patients with government-only insurance had the longest average LOS (20.30 days), followed by those with both government + private insurance (19.9 days) and private insurance patients (16.88 days). Uninsured patients had the shortest LOS (12.43 days), possibly due to resource limitations leading to earlier discharges or less intensive post-injury care. While shorter LOS among uninsured patients might reflect less severe injuries, it raises concerns about potential undertreatment or inadequate follow-up care. Previous studies have similarly linked longer LOS with complications such as infections or slower recovery, while uninsured patients often face poorer long-term outcomes due to limited resource utilization during hospitalization [17,18]. Future studies that incorporate injury severity and comorbidities are essential to confirm these findings and contextualize the LOS differences.

Although SSI rates did not differ significantly by insurance status (p = 0.847), uninsured patients had the highest rates (12.5%), warranting further investigation. The shorter LOS of uninsured patients may hinder the detection of infections, as many SSIs develop after discharge [18,19]. Emergency Medical Treatment and Labor Act (EMTALA) provisions may mitigate disparities in infection rates by ensuring initial care, but gaps in long-term follow-up for uninsured patients remain a concern [20]. This finding may underscore the need for improved outpatient follow-up care to prevent missed complications.

Our findings revealed no significant differences in operative management by insurance status, contrasting with prior studies suggesting that uninsured patients are less likely to receive surgery [20]. One explanation may be that the patients in our study did not present with injuries requiring operative intervention. The absence of significant differences in operative management may also reflect equal access, or lack thereof, to acute surgical care in our trauma center, though further studies are needed to assess broader trends.

Uninsured patients exhibited the highest mortality rates (20.2%), compared to lower rates for government-insured (9.9%), private-insured (14.9%), and combined insurance groups (16.7%). These differences highlight a possible protective role of government insurance in trauma settings, which ensures access to critical services without financial barriers [21]. The higher mortality among uninsured patients raises concerns about systemic inequities, including potential delays in seeking care, reduced resource allocation, and limited post-discharge support, all of which may be associated with unaffordability. Additionally, factors like healthcare provider implicit bias or even intended compassion for decreasing financial burden on patients may also have a role in these observations. Addressing these disparities will require a multifaceted approach, including expanding insurance coverage and implementing targeted community-based follow-up programs for uninsured patients.

Our findings align with studies from the mainland US, where uninsured patients also experience shorter LOS and worse outcomes [17,18]. These results emphasize the systemic healthcare disparities in Puerto Rico, exacerbated by its resource-constrained healthcare system. Efforts to expand public insurance coverage and enhance access to healthcare services are critical to mitigating these discrepancies. Targeted interventions, such as mobile health units or telemedicine follow-up programs, could address barriers faced by uninsured and underinsured populations, improving long-term recovery and survival outcomes.

This study has several strengths. The relatively large cohort of 1,165 patients provides sufficient statistical power to evaluate patterns in CMF trauma and allows for meaningful comparisons between insurance groups. Additionally, all patients were treated at a single, centralized trauma center, which ensures relatively consistent clinical protocols, documentation practices, and imaging evaluation across cases. The study also evaluates clearly defined clinical outcomes, including LOS, operative management, SSI, and survival. Furthermore, the study is conducted within the unique healthcare context of Puerto Rico, where reliance on public insurance programs is high, providing an opportunity to examine trauma outcomes within a distinct insurance landscape.

Several limitations should also be considered when interpreting the findings. The retrospective design may introduce biases related to the accuracy and completeness of medical records; additionally, findings should be interpreted as associations rather than causal relationships. In addition, the analysis did not include standardized injury severity metrics such as the Injury Severity Score, which may influence comparisons of outcomes between insurance groups and could result in differences that partially reflect variations in injury severity rather than insurance status. Importantly, there was limited data available on base health status and other socioeconomic variables of patients, preventing proper adjustment for potential clinical confounders. Additionally, although insurance status was obtained from the trauma registry, misclassification is possible in patients with multiple or overlapping insurance plans.

Future research should adopt a prospective, multicenter design to evaluate the impact of socioeconomic factors, comorbidities, and injury severity on trauma outcomes. Such efforts will provide a clearer understanding of healthcare disparities and inform equitable trauma care policies.

## Conclusions

This study demonstrates that insurance coverage significantly impacts outcomes for CMF trauma patients in Puerto Rico. Uninsured patients had shorter hospital stays and higher mortality rates, while insured patients, particularly those with government-only insurance, showed longer stays and better survival outcomes. These findings highlight the need for further research into the factors underlying these disparities, including systemic barriers to care and limitations in insurance coverage. Efforts to reduce the uninsured population and improve healthcare accessibility are essential to mitigating trauma outcome disparities.

## Appendices

Appendix A 

**Table 3:** ICD-10 codes for craniomaxillofacial trauma

ICD-10 code	Description
S00.4	Superficial injury of the ear
S00.8	Superficial injury of other parts of the head
S00.9	Superficial injury of the unspecified part of the head
S01.0	Open wound of the scalp
S01.1	Open wound of the eyelid and periocular area
S01.2	Open wound of the nose
S01.3	Open wound of the ear
S01.4	Open wound of the cheek and temporomandibular area
S01.5	Open wound of the lip and oral cavity
S01.8	Open wound on the other part of the head
S01.9	Open wound of the unspecified part of the head
S02.0	Fracture of the skull vault
S02.1	Fracture of the base of the skull
S02.2	Fracture of the nasal bones
S02.3	Fracture of the orbital floor or the orbital wall
S02.4	Fracture of the malar, maxillary, and zygomatic bones
S02.41-S02.42	LeFort fractures and pterygoid plate fractures
S02.5	Fracture of a tooth
S02.6	Fracture of the mandible
S02.8	Fracture of the other skull and facial bones
S02.9	Unspecified skull or facial fracture
S03.0	Dislocation of the jaw
S03.2	Dislocation of a tooth
S05.1-S05.9	Injuries to the eye and orbit
S09.9	Unspecified injury of the head/face
